# A Cross-Sectional Study Investigating Urinary Levels of Nerve Growth Factor and Its Precursor in Patients with Multiple Sclerosis

**DOI:** 10.3390/jcm15218341

**Published:** 2026-10-26

**Authors:** Viviana Nociti, Riccardo Bientinesi, Alessandra Cicia, Simone Coluzzi, Bernardo Rocco, Massimiliano Mirabella, Fiorella Miceli, Emilio Sacco, Diego Currò

**Affiliations:** 1 Unità Sclerosi Multipla, Fondazione Policlinico Universitario Agostino Gemelli IRCCS, 00168 Rome, Italy; 2 Centro di Ricerca per la Sclerosi Multipla “Anna Paola Batocchi”, Dipartimento di Neuroscienze, Università Cattolica del Sacro Cuore, 00168 Rome, Italy; 3 Urologia, Ospedale Mater Olbia, Fondazione Policlinico Universitario Agostino Gemelli IRCCS, 07026 Olbia, Italy; 4 Dipartimento di Medicina e Chirurgia Traslazionale, Sezione di Urologia e Nefrologia, Università Cattolica del Sacro Cuore, 00168 Rome, Italy; 5 Clinica Urologica, Dipartimento di Scienze Mediche e Chirurgiche, Fondazione Policlinico Universitario Agostino Gemelli IRCCS, 00168 Rome, Italy; 6 Dipartimento di Medicina e Chirurgia Traslazionale, Sezione di Farmacologia, Università Cattolica del Sacro Cuore, 00168 Rome, Italy; 7 Urologia, Ospedale Isola Tiberina—Gemelli Isola, 00186 Rome, Italy

**Keywords:** multiple sclerosis, overactive bladder, nerve growth factor, urinary biomarker

## Abstract

**Background/Objectives:** As multiple sclerosis (MS) progresses, most patients develop lower urinary tract symptoms (LUTS) indicative of an overactive bladder (OAB), primarily due to neurogenic detrusor overactivity, which is the most common abnormality identified during urodynamic testing. Therefore, biomarkers for OAB would be valuable additions to diagnostic tools. This study aimed to compare urinary levels of nerve growth factor (NGF) and its precursor, pro-NGF, in MS patients with and without OAB. **Methods:** We enrolled patients with relapsing MS (RMS), some exhibiting LUTS suggestive of OAB, along with healthy controls (HCs). Urine samples were collected, and NGF and pro-NGF levels were measured using ELISA kits. **Results:** Neither an ELISA kit specific for NGF nor a kit recognizing both NGF and pro-NGF detected measurable levels of these proteins. However, an ELISA kit selective for pro-NGF detected similarly low concentrations of this protein in a few samples from each group. **Conclusions:** Our ELISA results, measuring urinary NGF and pro-NGF levels in MS patients and HCs, are consistent with other studies that failed to detect these proteins in the urine of patients with OAB of various etiologies or in HCs. It is possible that urinary levels of NGF and pro-NGF are so low that the detection limits of ELISA kits and other available immunoassays are insufficient for their reliable measurement.

## 1. Introduction

Multiple sclerosis (MS) is a chronic inflammatory and degenerative demyelinating disease of the central nervous system (CNS) with a multifactorial etiology, characterized by broad inter- and intra-individual heterogeneity [1]. It affects 50 to 300 individuals per 100,000 people, is commonly diagnosed in young adults aged 20 to 40 years, and is three to four times more prevalent in women than in men [1]. Up to 90% of patients with MS develop lower urinary tract dysfunction (LUTD) within 18 years of their diagnosis [2,3]. A 2005 survey of nearly 10,000 MS patients conducted by the North American Research Committee on Multiple Sclerosis found that 65% of participants experienced one or more moderate-to-severe urinary symptoms, as well as bladder and sexual disorders [4,5,6]. The presentation and severity of lower urinary tract symptoms (LUTS) can vary considerably among patients with MS due to multifocal and diffuse involvement of the CNS. LUTS in patients with MS may include urinary frequency, urgency, and urge incontinence, which can range in severity [7]. These symptoms may be accompanied by incomplete bladder emptying and/or hesitancy. LUTS significantly impairs patients’ quality of life, with approximately 70% of patients reporting a moderate-to-severe impact [8]. Moreover, these symptoms carry a high risk of compromising the integrity of the upper urinary tract [9].

A systematic review of 12 studies involving 1524 MS patients reported that 53% had neurogenic detrusor overactivity (NDO), 43% had detrusor-sphincter dyssynergia, and 12% had bladder atony [10]. Although comprehensive urodynamic testing remains the standard approach for neuro-urological diagnosis, recent research has explored novel, non-invasive diagnostic methods. Among these, urinary nerve growth factor (NGF) has emerged as a potential biomarker for neurogenic bladder conditions [11]. NGF is a key regulator of neuronal plasticity within the nerve pathways involved in urination [12]. It is released into the bladder by the smooth muscle and urothelium [12], and bladder wall stretching, common in obstructed bladders, stimulates its production [13]. NGF modulates neurotransmitter release, induces synaptic reorganization, and influences afferent neuron excitability [14]. Additionally, it sensitizes most afferent nerve endings in the bladder, thereby promoting neurogenic inflammation [15]. Studies have also demonstrated that NGF contributes to the development of hypersensitivity, urinary urgency, and detrusor overactivity (DO) in animal models [16]. Together with other experimental findings, these results suggest that NGF plays a pivotal role in triggering neuronal changes that lead to neurogenic LUTD (NLUTD) and overactive bladder (OAB).

The use of NGF levels in urine as a potential biomarker for OAB has been proposed [17]; however, its specificity for OAB remains questionable [18]. The literature on urinary NGF and OAB is conflicting, likely because different ELISA kits were primarily used in the studies, none of which were validated for measuring NGF in urine. NGF urinary levels reported in these studies were generally very low, often near or below the lowest point of the assay standard curves, raising concerns about the reliability of the measurements. Furthermore, the most commonly used ELISA kit, the NGF Emax Immunoassay (Promega, Madison, WI, USA), was subsequently withdrawn from the market due to technical issues, casting doubt on the validity of some positive results reported in these studies [19].

Two recent studies have reported elevated levels of NGF in the urine of MS patients and NLUTD [20,21]. However, neither study included a control group of MS patients without NLUTD. Therefore, comparative studies that include a control group of MS patients without NLUTD are essential to evaluate NGF as a potential urinary biomarker for NLUTD in this population. Our study aimed primarily to compare urinary NGF levels between MS patients with OAB and those without OAB. The secondary objective was to assess whether there are differences in urinary levels of pro-NGF, the precursor of NGF, among these groups. An imbalance between NGF and pro-NGF has been observed in older individuals and patients with neurodegenerative diseases [22,23,24], which may have significant implications. Pro-NGF can induce either apoptosis or cell survival, whereas NGF primarily promotes cell survival [24,25]. The biological rationale for examining both mature NGF and pro-NGF is supported by the compartmentalized processing of the NGF precursor. Although pro-NGF can be cleaved into mature NGF, the two forms exhibit different receptor affinities and may elicit distinct biological effects [24]. Consequently, a measurement reported as “NGF” is not necessarily comparable across assays unless antibody specificity and cross-reactivity are well characterized. This distinction is particularly important in urine, where factors such as the low protein concentration and matrix effects can influence the apparent analyte levels. Therefore, the present study was designed not only to compare symptomatic and asymptomatic patients but also to technically assess whether commercially available immunoassays can reproducibly quantify urinary NGF-related proteins in this clinical context.

## 2. Materials and Methods

The study was approved by the Institutional Ethics Committee of the Catholic University of Rome, Italy (Protocol ID 4653).

Between November 2023 and December 2025, we enrolled patients with relapsing MS (RMS) who were referred to the MS Center of the “Agostino Gemelli” Hospital in Rome and met the following inclusion criteria: (1) a diagnosis of RMS according to the 2017 revisions of the McDonald criteria [26]; (2) treatment with intravenous ocrelizumab every six months; (3) presence or absence of LUTS suggestive of OAB, as determined by urological evaluation and patient-reported outcome measures; and (4) signed informed consent. The exclusion criteria were: (1) patients experiencing clinical and/or neuro-radiological relapse at the time of enrollment or within the preceding three months (due to potential increases in neurotrophic factors under these conditions); (2) patients with ongoing infectious or inflammatory processes (for the same reason); (3) pregnant women; (4) patients who had received symptomatic therapy for urinary disorders, including urological antimuscarinics, in the three months prior to sample collection, or antibiotics for urinary tract infections at the time of urine sample provision; and (5) failure to provide signed informed consent.

This cross-sectional study consecutively enrolled eligible participants into three groups: patients with RMS who reported symptoms of OAB (LUTS+ patients), patients with RMS without OAB symptoms (LUTS− patients), and healthy controls (HCs) matched for age and sex. To ensure sample homogeneity, only patients receiving ocrelizumab treatment were included. Healthy controls matched to age and sex were recruited from hospital staff volunteers. Urine samples were collected during routine neurological follow-up visits, aliquoted into 15 mL Falcon conical tubes, centrifuged at 2000× *g* for 15 min at 4 °C to remove pellets, and stored at −80 °C. During these visits, data on age, sex, body mass index (BMI), disease duration, duration of ocrelizumab treatment, and clinical and neuroradiological parameters were also collected. Neurological disability was assessed at the time of urine sampling using the Expanded Disability Status Scale (EDSS). Patient-reported outcome measures included the International Consultation on Incontinence Questionnaire-Overactive Bladder (ICIQ-OAB), the International Consultation on Incontinence Questionnaire-Urinary Incontinence Short Form (ICIQ-UI SF), and the Incontinence Quality of Life (I-QoL) questionnaire.

Our primary objective was to demonstrate a statistically significant difference in urinary NGF levels between MS patients with and without LUTS. The literature on NGF in MS reports a mean ± SEM urinary NGF concentration of approximately 4.4 ± 0.6 pg/mL in healthy volunteers [21]. According to published data, patients with MS and NDO exhibit urinary NGF levels approximately 10.3 times higher than those of patients with other neurological disorders and NDO [20]. Thus, elevated urinary NGF concentrations appear to be a specific characteristic of patients with NDO associated with MS. However, increased urinary NGF levels may also represent a general feature of MS. Therefore, we hypothesized that urinary NGF levels would be 1.5 times higher in LUTS+ MS patients compared to LUTS− MS patients. Assuming equal variance between groups, a two-tailed *t*-test with 90% power, a 5% significance level, and a coefficient of variation of 0.65 indicated that 47 subjects per group were required to detect this difference. We planned to conduct an interim analysis after enrolling at least 10 patients per arm to evaluate the validity of our hypothesis and, if necessary, reassess the sample size.

During the assay preparation, the urine samples were thawed at room temperature, vortexed, and centrifuged again at 4 °C to remove any remaining impurities. The urine samples were then used either directly or after concentration by ultrafiltration with Millipore Amicon Ultra-4 centrifugal filter devices (Merck KGaA, Darmstadt, Germany), following the manufacturer’s instructions. Filters with a 10 kDa molecular weight cut-off were selected because the native mature NGF is a homodimer with an approximate molecular weight of 27 kDa. This ultrafiltration method reduces 4 mL of urine to 40–60 μL. The concentrated samples were then rapidly dried using a Savant SpeedVac vacuum concentrator (Thermo Fisher Scientific, Waltham, MA, USA). The resulting pellets were reconstituted in 250 μL of 1× buffer at the time of the assay preparation, achieving a sixteen-fold concentration. Three ELISA kits were used to measure urinary levels of NGF and/or pro-NGF: the Human beta-NGF DuoSet ELISA kit (R&D Systems, Minneapolis, MN, USA); the Nerve Growth Factor (Beta-NGF) Human Rapid™ ELISA kit; and the Nerve Growth Factor pro- (pro-NGF) Human Rapid™ ELISA kit (Biosensis, Thebarton, SA, Australia). The latter two kits selectively measure either the mature form of NGF or its precursor, pro-NGF, respectively. The manufacturer reported detection limits of 2 pg/mL for NGF and <60 pg/mL for pro-NGF for these two kits. Of the various NGF ELISA kits available on the market, we initially selected the Human beta-NGF DuoSet ELISA kit from R&D Systems because it is well characterized, and the capture and detection antibodies used in this kit have been shown to recognize both pro-NGF and the mature form of NGF [27,28]. This ensured that total NGF-like immunoreactivity (NGF-LI; i.e., both NGF and pro-NGF) levels could be measured in the samples. An important technical consideration influenced our choice: this kit provides an assay buffer that is 10 times more concentrated than required. Therefore, the recommended 1× buffer was obtained by diluting the 10× solution 1:10 for each sample. Specifically, 25 µL of the 10× buffer solution was added to 225 µL of each sample. Then, 100 µL of the resulting solution was used per well in duplicate for each sample. To ensure comparable conditions between the standard curve and the samples, all standard curve solutions were prepared using an assay buffer made by diluting the 10× assay buffer with artificial urine, which was prepared as described by Sarigul et al. [29]. Following the kit protocol, a standard curve with mature NGF was prepared by starting at 2 ng/mL and performing serial 1:2 dilutions down to 31.25 pg/mL. We extended the standard curve by adding three additional points through further 1:2 dilutions of the 31.25 pg/mL solution, reaching a concentration of 3.9 pg/mL. This resulted in a ten-point standard curve instead of the recommended seven. The other two kits from Biosensis are well-established ELISA kits that allow differentiation between NGF and pro-NGF levels in samples. Optical density was measured at 450 nm using a microplate absorbance reader (iMark; Bio-Rad Laboratories Inc., Hercules, CA, USA) with a reading time of 15 s. Each sample was analyzed in duplicate.

## 3. Results

We conducted an interim analysis after enrolling 11 patients with LUTS+ MS, 12 patients with LUTS− MS, and 11 HCs. In the urine samples tested, no optical absorbance exceeding that of the blank was detected using the Human beta-NGF DuoSet ELISA kit from R&D Systems. Consequently, we concentrated the urine samples using Millipore Amicon Ultra-4 centrifugal filters, followed by vacuum drying, to increase NGF-LI levels in the assay wells. However, even in these concentrated samples, the mean optical absorbance in no sample exceeded the mean blank value when measured with the same ELISA kit.

We then tested the concentrated samples using the Nerve Growth Factor (Beta-NGF) Human Rapid™ ELISA kit and the Nerve Growth Factor pro- (proNGF) Human Rapid™ ELISA kit from Biosensis, which specifically measure the levels of the mature form of NGF and its precursor, respectively. However, even with the kit specific for mature NGF, no protein levels were detected above the assay’s detection limit. In contrast, the Biosensis kit designed to detect pro-NGF measured very low levels of this protein in two out of 11 urine samples from LUTS+ MS patients, four out of 12 samples from LUTS− MS patients, and three out of 11 samples from HCs. The respective mean values ± standard deviation were 5.5 ± 6.5 pg/mL (*n* = 2), 8.2 ± 1.9 pg/mL (*n* = 4), and 13.8 ± 3.7 pg/mL (*n* = 3). The standard curves for the three ELISA kits were satisfactory (Supplementary Materials, Figure S1). Additionally, the two Biosensis ELISA kits included quality control samples, and the measured levels of these controls were within the ranges specified by the manufacturer in the assay protocols.

Based on the preliminary data obtained from the interim analysis, we decided to suspend patient enrollment and terminate the study with the sample size achieved up to that point. Consequently, a total of 23 patients with RMS and 11 HCs were enrolled in the study. Table 1 summarizes the baseline demographic and clinical characteristics of the study cohort. No significant differences in age or BMI were observed between patients and HCs. Additionally, no significant differences were found in baseline age, BMI, EDSS score, disease duration, or ocrelizumab exposure period between the LUTS+ and LUTS− groups.

## 4. Discussion

As the disease progresses, between 34% and 99% of patients with MS experience urinary symptoms or signs due to NLUTD [30]. The most frequently reported symptoms are urinary frequency and urgency, which can vary in severity [30]. Urinary urgency causes urinary incontinence in 27% to 66% of patients [3]. NDO is the most common abnormality identified during urodynamic testing in patients with MS and LUTS [10]. However, these examinations are invasive, time-consuming, and costly. Therefore, the availability of diagnostic, monitoring, and treatment response biomarkers would be highly beneficial. The clinical value of a urinary biomarker would be greatest if it provided information beyond symptoms and basic functional assessments. Currently, the evaluation of MS-related NLUTD remains primarily clinical and urodynamic: symptom scores, bladder diaries, post-void residual volume measurements, uroflowmetry, and, when indicated, multichannel or video-urodynamics are used to define the phenotype and identify patients at risk of high-pressure storage, inefficient emptying, or upper-tract complications. Therefore, a biomarker should be evaluated against a prespecified reference standard, ideally incorporating urodynamic phenotype and longitudinal outcomes, rather than relying solely on OAB symptoms. This distinction is important because urgency and frequency do not uniquely identify NDO, and patients with similar symptoms may exhibit different pressure–flow patterns and varying risks to the upper urinary tract [2,3,11,12].

NGF is one of the most extensively studied molecules as a potential biomarker for OAB. However, a recent meta-analysis concluded that NGF cannot be used as an OAB biomarker due to its lack of specificity [31]. Gamper and Viereck [32] criticized the findings of this meta-analysis. First, they noted that the lack of specificity had already been reported by Seth et al. [18]. More importantly, they highlighted the technical issues with the NGF Emax Immunoassay (Promega, Madison, WI, USA), the ELISA kit used in 13 of the 17 studies included in the meta-analysis. This ELISA kit, withdrawn from the market in May 2014, has been shown to nonspecifically detect other urinary components, particularly human IgG [19]. Consequently, Gamper et al. [19] strongly recommended that results obtained primarily using this ELISA kit be validated with alternative assays.

Several studies have investigated urinary levels of NGF in patients with NLUTD. Of the seven studies on urinary NGF levels included in a recent systematic review of urinary biomarkers in NLUTD [11], four reported higher NGF levels in patients with NLUTD compared to healthy controls [20,33,34,35]. Notably, three of these four studies employed the Promega ELISA kit, which raises concerns about the reliability of the results [33,34,35]. The fourth study utilized a RayBiotech (Peachtree Corners, GA, USA) ELISA kit [20]; however, the authors directly pipetted urine samples into the plate wells without dilution, creating different test conditions between the sample wells and the standard curve wells, which contained assay buffer [20]. For ELISA, it is generally recommended that samples be diluted at least 1:2 with assay buffer to minimize nonspecific binding. This latter study is one of only two in the literature that enrolled MS patients. It reported that patients with MS and NDO exhibited higher urinary and/or serum NGF levels than patients with spina bifida or spinal cord injuries and NDO [20]. The other study, which was not included in the aforementioned systematic review and used the Promega ELISA kit, also found elevated urinary NGF levels in MS patients with NDO compared to HCs [21]. Using the Duoset ELISA kit from R&D Systems, which detects both NGF and pro-NGF, and the beta-NGF Rapid™ ELISA kit from Biosensis, which specifically detects NGF, we were unable to detect any NGF-LI in urine samples from any patient or HC enrolled in our study, whether the samples were plain or concentrated sixteen fold. However, using the Biosensis Pro-NGF Rapid™ ELISA kit, which selectively detects pro-NGF, we found very low levels of NGF-LI in concentrated urine samples from six MS patients and three HCs. The mean NGF-LI levels measured across the three groups appeared similar. We do not have a clear explanation for the absence of NGF-LI detection using the R&D Systems ELISA kit, although NGF-LI was detected in some samples using the Biosensis pro-NGF ELISA kit. The differing cross-reactivities and affinities of the antibodies used in the two kits likely account for these discrepancies. In any case, the absorbance values measured for these urine samples were only slightly higher than those of the blanks, falling within the lower portion of the lower plateau of the sigmoid standard curve. Consequently, the measured pro-NGF levels are questionable because reliable quantification in an assay depends on values that fall within, or as close as possible to, the linear portion of the sigmoid curve. Other authors have also reported that NGF is undetectable in the urine of patients with NLUTD or HCs. Regauer et al. [36] were unable to detect any NGF in urine from patients with bladder pain syndrome/interstitial cystitis or OAB, or in HC samples, using the Multi-Spot 96 4-Spot Prototype (Meso Scale Discovery, Rockville, MD, USA), a multiplex immunoassay based on electrochemiluminescence. Similarly, Krebs et al. [37] reported no NGF in urine samples from patients with NLUTD following spinal cord injuries and from HCs using the Human β-NGF Standard ABTS ELISA Development Kit (PeproTech Inc., London, UK). Colic et al. [38] found unquantifiable urinary concentrations of NGF in 96% of samples analyzed by ELISA (Merck, Darmstadt, Germany) and in 100% of samples analyzed by the Luminex platform (Thermo Fisher Scientific, Waltham, MA, USA), a technology combining ELISA principles with flow cytometry, in a pediatric population of patients with OAB or HCs. Therefore, our results indicate that NGF or pro-NGF levels in urine are very low, such that ELISA or other available immunoassays, with detection limits in the low pg/mL range, are not ideal for measuring these proteins. In any case, the very low levels of pro-NGF detected in some urine samples in our study do not appear to differ significantly between HCs and patients with MS, with or without NLUTD. The decision to halt recruitment was based on analytical considerations rather than evidence of equivalence between groups. Most observations fell below the detection range of the assays, and the few pro-NGF signals detected were limited to a subset of samples and located near the lower end of the standard curve. Under these conditions, conventional between-group hypothesis testing would have limited interpretability because the effective sample size depends on the number of quantifiable observations, not merely on the number of enrolled participants. Therefore, the findings should be reported as evidence of assay non-quantifiability in this cohort, rather than as definitive proof that urinary NGF or pro-NGF is absent or biologically equivalent across groups.

Regarding future research directions, initial studies should first establish analytical validity before assessing clinical utility. A multicenter protocol should include urine samples from patients with urodynamically confirmed NDO; MS patients with LUTS but without NDO; MS patients without LUTS; neurological controls with NDO; and HCs. Samples should be collected prospectively at standardized time points and analyzed using blinded replicates. Orthogonal confirmation methods, such as targeted immunoassays with validated urine-matrix recovery, immunoblotting, or targeted mass spectrometry, would help differentiate true low-level analyte detection from nonspecific immunoreactivity. Concurrent measurement of urinary brain-derived neurotrophic factor alongside inflammatory or epithelial markers may clarify whether the neurotrophin signal reflects neuronal remodeling, urothelial irritation, infection, or a broader inflammatory phenotype [11,20,21,30].

Because urinary protein levels are influenced by dilution, pre-analytical standardization is essential when comparing participants. Future implementation of this protocol should prespecify factors such as collection time, interval since the last void, hydration status, urine volume, storage duration, and normalization strategy, such as urinary creatinine. These variables are important because concentration-based results may reflect urine dilution rather than changes in bladder biology. In the present exploratory study, identical collection and storage procedures were employed across all groups; however, we chose not to normalize pro-NGF levels in our samples to urinary creatinine because only a few samples had detectable pro-NGF, and the pro-NGF levels were questionable, as their absorbance values were close to those of the assay blanks [11,22].

The most informative study design would be longitudinal and linked to treatment. Urine samples should be collected before and after pharmacological interventions, catheterization changes, or rehabilitation, alongside simultaneous assessments of symptoms, bladder diaries, renal function, and urodynamics. The biomarker should then be evaluated for predefined outcomes, such as diagnostic discrimination, prediction of treatment response, and detection of clinically meaningful deterioration, using receiver operating characteristic analysis, calibration, and external validation. For example, one study suggests that urinary NGF levels may change following botulinum toxin treatment; however, reported diagnostic performance and correlations with urodynamic outcomes have been inconsistent [21]. Therefore, a clinically useful assay cannot be inferred from a statistically significant concentration change alone.

If analytical sensitivity and specificity can be established, urinary NGF-related measurements could ultimately support risk-stratified surveillance, reduce unnecessary invasive testing in selected low-risk patients, and identify those requiring earlier urodynamic evaluation. Conversely, until these steps are completed, the practical application of the current findings remains primarily methodological: results below the assay’s detection range should not be interpreted as reliable negative biomarker outcomes, and commercially available NGF/pro-NGF ELISA values should not be compared across studies without careful consideration of antibody specificity, matrix validation, calibration range, normalization, and pre-analytical handling [11,19,28].

## 5. Conclusions

Our study did not detect measurable levels of NGF-LI in urine samples from MS patients, both with and without LUTS, or from HCs, even after concentrating the samples sixteen fold using two ELISA kits from different manufacturers. Using a third ELISA kit specific for pro-NGF, we detected very low levels of this protein in 26.5% of the samples, with similar results across all three groups. Other studies have also reported undetectable NGF-LI levels in urine when using ELISA kits or other immunoassays. Therefore, the detection limits of most commercially available immunoassays may be insufficient for accurately measuring urinary NGF and pro-NGF levels.

These results also delineate the limitations of the current study. The investigation was exploratory, cross-sectional, single-center, and involved a small sample size; only a minority of pro-NGF measurements exceeded the relevant assay signal threshold, and all participants were receiving the same disease-modifying treatment. Therefore, the findings should not be generalized to untreated MS, progressive MS, other neurological disorders, or patients with urodynamically confirmed NDO. Instead, these results underscore the need for assay-specific validation and adequately powered, longitudinal studies before urinary NGF or pro-NGF can be integrated into routine diagnostic or monitoring protocols.

## Figures and Tables

**Table 1:** **Demographic and clinical characteristics of the study cohort.** Continuous variables are expressed as mean ± standard deviation or median with interquartile range when appropriate, and categorical variables as n with %. Comparisons between LUTS+, LUTS− and HC groups were performed using one-way ANOVA for continuous variables and the chi-square test for categorical variables. Comparisons between LUTS+ and LUTS− groups were performed using the t-test or Mann–Whitney U-test, as appropriate.

	LUTS+ (*n* = 11)	LUTS− (*n* = 12)	HCs (*n* = 11)	*p* Value
Age, years—Mean ± SD	38.1 ± 8.6	36.1 ± 8.7	37.2 ± 9.8	0.48
Sex, female—n (%)	6 (54.5)	5 (41)	5 (45.5)	0.82
BMI—Mean ± SD	22.3 ± 0.9	22.2 ± 1.3	22 ± 1.0	0.74
Disease duration, years—Mean ± SD	6.6 ± 4.9	4.8 ± 4.9		0.85
EDSS score—Median (IQR)	3 (1.5)	2 (1.5)		0.19
Duration of ocrelizumab treatment, years—Mean ± SD	2.9 ± 1.3	2.3 ± 1.1		0.23
ICIQ-OAB score—Median (IQR)	13 (2.5)	0 (1)	0	
ICIQ-UI SF score—Median (IQR)	14 (3.5)	0 (1)	0	
I-QoL score—Median (IQR)	52 (8)	95 (7.25)		<0.001

Abbreviations: BMI, Body Mass Index; EDSS, Expanded Disability Status Scale; HCs, Healthy Controls; ICIQ-OAB, International Consultation on Incontinence Questionnaire-Overactive Bladder; ICIQ-UI SF, International Consultation on Incontinence Questionnaire-Urinary Incontinence Short Form; I-QoL, Incontinence-Quality of Life; IQR, Interquartile Range; LUTS, lower urinary tract symptoms; MS, Multiple Sclerosis; SD, Standard Deviation. Higher ICIQ scores indicate greater burden of symptoms, while higher I-QoL scores indicate better quality of life related to the urinary tract.

## Data Availability

The original data regarding the standard curves for the ELISA kits used in this study are provided in the Supplementary Materials. Further inquiries can be directed to the corresponding authors.

## Supplementary Materials

The following supporting information can be downloaded at: https://www.mdpi.com/article/10.3390/jcm15218341/s1, Figure S1: ELISA kits’ standard curves.
