# Supplementary material for: A Cross-Sectional Study Investigating Urinary Levels of Nerve Growth Factor and Its Precursor in Patients with Multiple Sclerosis
Source: J Clin Med. 2026 Oct 26;15(21):8341. doi: 10.3390/jcm15218341 (PMC13763615; doi:10.3390/jcm15218341)
Supplement: Supplementary file 1 [file jcm-15-08341-s001.zip › jcm-4519135-supplementary.pdf]

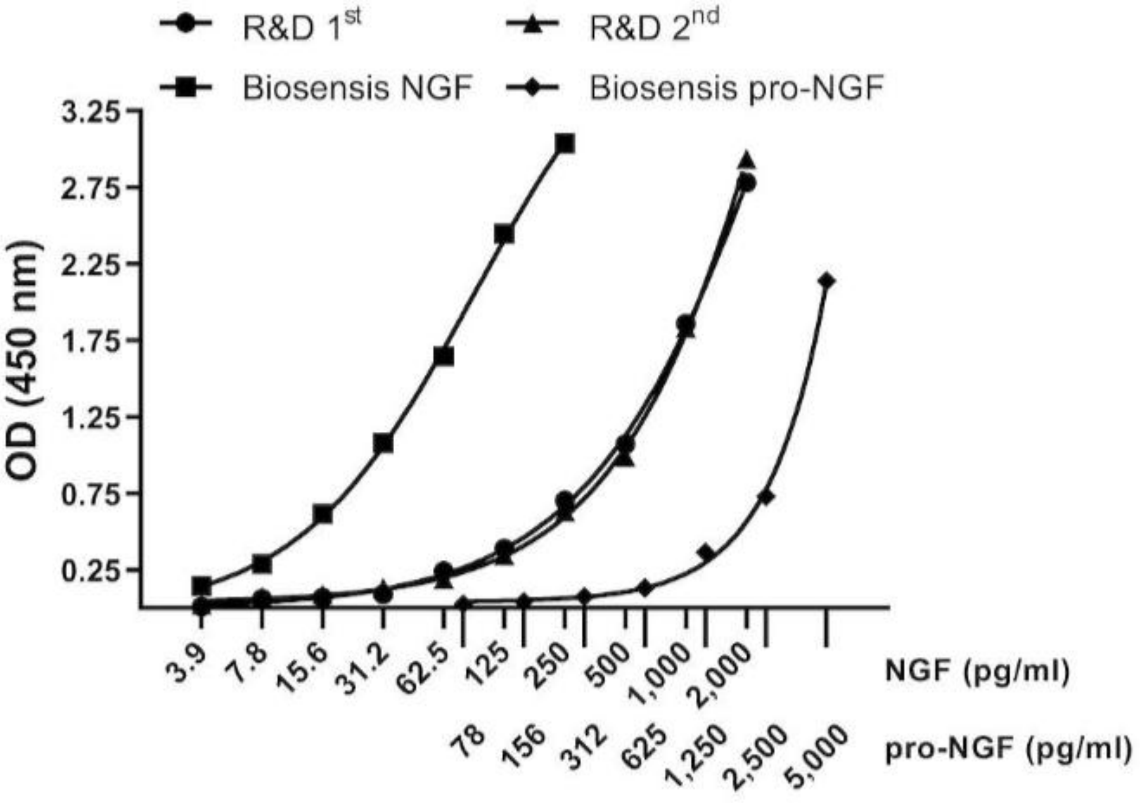

Figure S1. ELISA kits' standard curves. The standard curves shown for NGF or pro-NGF were obtained using the following ELISA kits: the "Human beta-NGF DuoSet ELISA" kit (R&D Systems, Minneapolis, MN, USA) and the "Nerve Growth Factor (Beta-NGF) Human Rapid™ ELISA" and "Nerve Growth Factor pro-(proNGF) Human Rapid™ ELISA" kits (Biosensis, Thebarton, South Australia, Australia). The GraphPad Prism program (GraphPad Software, San Diego, CA, USA) was used to fit the sigmoidal four parameter logistic (4-PL) curves.

Two curves are shown for the R&D Systems kit. The first was made using an assay buffer solution prepared by diluting the 10x assay buffer 1:10 with artificial urine prepared as described by Sarigul et al. (2019) [1]. The second was made using 1x assay buffer.

References

1. Sarigul N, Korkmaz F, Kurultak İ. A New Artificial Urine Protocol to Better Imitate Human Urine. Sci Rep. 2019 Dec 27;9(1):20159. doi: 10.1038/s41598-019-56693-4. PMID: 31882896; PMCID: PMC6934465.
